# The complete chloroplast genome sequence of *Patrinia monandra:* a traditional Chinese medicinal herb

**DOI:** 10.1080/23802359.2022.2098855

**Published:** 2022-08-28

**Authors:** Xinhong Wang, Lulu Li, Xiaojun Ma, He Su, Jing Shu

**Affiliations:** a College of Forestry Engineering, Shandong Agriculture and Engineering University, Jinan, China; b The Second Clinical College of Guangzhou University of Chinese Medicine, Guangzhou, China

**Keywords:** Chloroplast genome, *Patrinia monandra*, phylogenetic analyses

## Abstract

*Patrinia monandra* C. B. Clarke is traditionally used for the treatment of inflammation, typhoid fever, injuries due to falls, abdominal pain, malaria, and acute appendicitis ulcers in China and Korea. In this study, the complete chloroplast genome of *P. monandra* was sequenced using the Illumina HiSeq X-Ten platform. It had a circular shape and a length of 158,940 bp, with 38.51% GC content. It contained a large single-copy region of 87,641 bp, a small single-copy region of 12,807 bp, and two inverted repeat regions of 29,246 bp. In total, 128 different genes, including 83 protein-coding genes, 37 distinct tRNA genes, and eight rRNA genes, were identified. Maximum-likelihood phylogenomic analysis showed that *P. monandra* is closely related to *Patrinia scabiosifolia* and *Patrinia villosa* in the Valerianaceae family. There were 4535 variable sites, 157,354 conserved sites, and 3085 singleton sites in the six *Patrinia* chloroplast genomes.

*Patrinia* is a perennial (sometimes biennial) genus of the Valerianaceae family. Most of its species have been used for medicinal purposes (named ‘Bai Jiang Cao’) in China. The dry stems and roots of *Patrinia* have been used for the treatment of inflammation, typhoid fever, injuries due to falls, abdominal pain, malaria, and acute appendicitis in traditional Chinese and Korean medicine for thousands of years. Twenty species of *Patrinia* have been reported in East Asia and North America. In China, over 10 species, three subspecies and two varieties have been identified (He et al. 2017). *Patrinia monandra C. B. Clarke* [Fl. Brit. India 3:210 (1881)], which is named ‘Shao Rui Bai Jiang’ in Chinese, has been widely used medicinally for more than 2000 years.

The chaotic use of medicinal materials is a common phenomenon due to the difficulty in morphological identification (Li et al. 2015). With the advancement of molecular techniques, the identification of traditional Chinese medicine (TCM) has been successfully applied to identify medicinal plants (de Boer et al. 2015). Owing to its maternal inheritance and conserved structure, the chloroplast genome has been commonly used for DNA barcoding studies in TCM. The Chinese Pharmacopoeia Commission and the British Pharmacopoeia Commission have incorporated DNA barcoding into the methods list for the authentic identification of herbal medicine (Zhang et al. 2020).

In this study, the complete chloroplast genome of *P. monandra* has been reported for the first time. The results of this study will provide more information for molecular identification and phylogenetic reconstruction of the genus *Patrinia*.

No ethical approval or permission was required for this study. The collection of wild plant samples in this study strictly abides by the regulations of the People's Republic of China on Nature Reserves. Fresh and healthy leaves of *P. monandra* were collected from Guangdong Province (23°43′25″ N, 115°13′25″ E) in China and immediately frozen in liquid nitrogen. Later, the samples were maintained at −80 °C. The voucher specimen was authenticated by Ph.D. He Su and deposited at The Second Clinical Medical College of Guangzhou University of Chinese Medicine (voucher number: 20190528-1; the person in charge of the collection: He Su; email: suhe@gzucm.edu.cn). Total DNA was extracted and purified using a previously published methodology (Chen et al. 2019). The extracted DNA was validated using 1% (w/v) agarose gel electrophoresis and NanoDrop spectrophotometer 2000. Paired-end (2 × 150 bp) sequencing was performed using the Illumina HiSeq X-Ten platform. Paired-end reads were qualitatively assessed and assembled using GetOrganelle (Jin et al. 2020). Annotation was performed using GeSeq (Tillich et al. 2017). The annotated genomic sequence was submitted to GenBank (https://www.ncbi.nlm.nih.gov/) under accession number OL875076.

The complete chloroplast genome of *P. monandra* has a circular shape of 158,940 bp in length, with 38.51% GC content. The genome consisted of a large single-copy region of 87,641 bp, a small single-copy region of 12,807 bp, and two inverted repeat regions of 29,246 bp; 128 different genes, including 83 protein-coding genes, 37 distinct tRNA genes, and eight rRNA genes were identified.

The chloroplast genomes of five Valerianaceae species, seven Caprifoliaceae species, and two Dipsacaceae species were obtained from NCBI GenBank database. Phylogenetic analyses were performed for all downloaded chloroplast genomes and the *P. monandra* chloroplast genome from this study. The 17 complete chloroplast sequences were aligned using MAFFT version 7.490 software (Katoh and Standley 2013), and a phylogenetic tree was constructed using MEGA11 (Tamura et al. 2021). The robustness of the topology was estimated with 1000 bootstrap replicates using the maximum-likelihood method (Tamura et al. 2004). The phylogenetic tree revealed that *P. monandra* is closely related to *P. scabiosifolia* and *P. villosa* in the Valerianaceae family (Figure 1).

**Figure 1.**
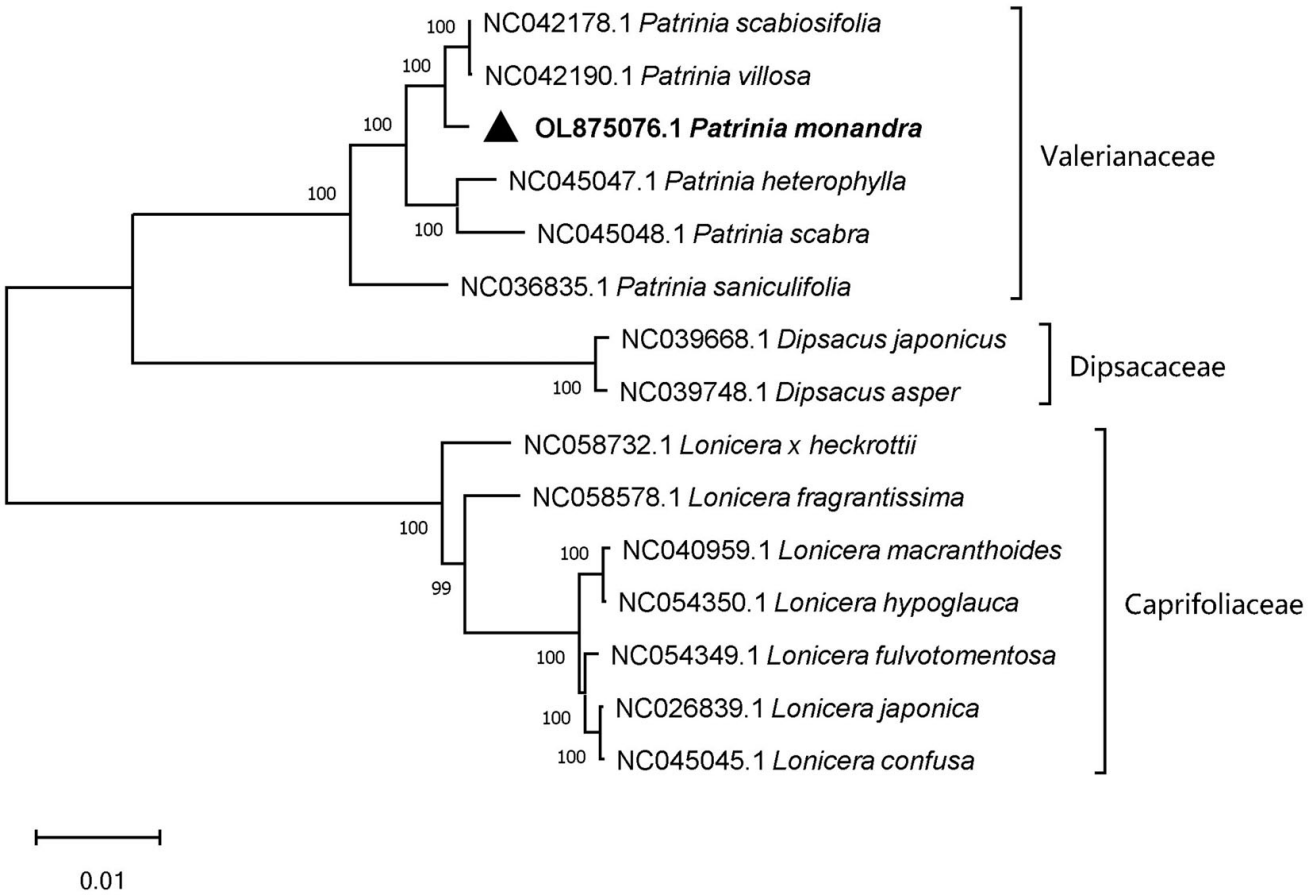
Phylogram of *P. monandra* and related species based on whole chloroplast genome sequences.

To predict the alternative DNA barcodes, highlight analysis was performed on six chloroplast genomes of the *Patrinia* species using MEGA11 (Tamura et al. 2021). There were 4535 variable sites, 157,354 conserved sites and 3085 singleton sites within the 185,620 bp region.

## Data Availability

The data that support the findings of this study are openly available at https://www.ncbi.nlm.nih.gov/. The complete chloroplast genome has been deposited in GenBank with accession number OL875076. The associated BioProject, SRA, and Bio-Sample numbers are PRJNA806379, SRX14155274, and SAMN25876042, respectively.
